# A web-based platform for the annotation and analysis of NAR-published databases

**DOI:** 10.1371/journal.pone.0293134

**Published:** 2023-10-23

**Authors:** Marcel Friedrichs, Cassandra Königs

**Affiliations:** Bioinformatics / Medical Informatics Department, Bielefeld University, Bielefeld, NRW, Germany; CCET: Chandigarh College of Engineering and Technology, INDIA

## Abstract

Biological databases are essential resources for life science research, but finding and selecting the most relevant and up-to-date databases can be challenging due to the large number and diversity of available databases. The Nucleic Acids Research (NAR) journal publishes annual database issues that provide a comprehensive list of databases in the molecular biology domain. However, the information provided by NAR is limited and sometimes does not reflect the current status and quality of the databases. In this article, we present a web-based platform for the annotation and analysis of NAR-published databases. The platform allows users to manually curate and enrich the NAR entries with additional information such as availability, downloadability, source code links, cross-references, and duplicates. Statistics and visualizations on various aspects of the database landscape, such as recency, status, category, and curation history are also provided. Currently, it contains a total of 2,246 database entries of which 2,025 are unique with the majority updated within the last five years. Around 75% of all databases are still available and more than half provide a download option. Cross references to Database Commons are available for 1,889 entries. The platform is freely available online at https://nardbstatus.kalis-amts.de and aims to help researchers in database selection and decision-making. It also provides insights into the current state and challenges of a subset of all databases in the life sciences.

## Introduction

Each year more biologic databases are created and added to the plethora of existing databases [1–3]. For researchers, this may pose a serious problem when selecting databases for a research project or analysis. It is becoming near impossible to know all databases that may be relevant and in addition, the quality or actuality of the included information is not always apparent. For example, Mubeen *et al.* analyzed the impact of pathway database choice on analysis results [4]. Duck *et al.* surveyed bioinformatics resources using text mining and have shown that only a few resources account for 47% of total usage and over 70% of resources are only mentioned once according to their results [5]. This imbalance results in important databases potentially not being discovered. Therefore, it is important to get a good overview of which databases exists for a specific task or field of research. Several projects exist trying to catalog and annotate as many databases or sometimes also tools as possible such as Database Commons [3], FAIRsharing [6], and bio.tools [7].

FAIRsharing [6] contains 2,031 different databases as of 13.06.2023 with the status ready, in development, uncertain, and deprecated. For each database information about the year of creation, website URL, publications, and when the database was last checked are provided.

bio.tools [7] contains 691 databases as of 13.06.2023 with the topic ‘Biological databases’, but this does not include all databases and several other filters are possible. Databases like DrugBank or KEGG for example, are not part of this number. There exist no databases only filter yet which is likely due to the fact that the website was originally developed to be a catalog of tools. Each database has information on publications, website URLs, versions, and GitHub links where available.

Database Commons [3] contains 5,904 different databases as of 13.06.2023. For each database it has information on the website URL, the last update of the database, availability, country, and publications. The availability annotation is done both manually and using an automated real-time check. People may register to curate existing and submit new entries with the history of who curated at what time-point visible for each entry. With nearly 6,000 entries the manual curation process is no trivial task. This is visible by the aforementioned curation history, for example, the entry with id 5954 was last curated in 2019 as of 25.09.2023. The same entry also shows obvious data errors, as entry 5954 has the name “Gene3D” but is actually “The Genomic Threading Database” with the real “Gene3D” database having the id 188. There are also duplicates such as the “APPRIS” database with ids 323 and 7795. Finally, the automated real-time availability check is often wrong, as the plain HTTP response code is not a detailed enough indicator of a database’s availability. If a database stops existing and the URL is redirected to another site, the response code will still remain as code 200. Sometimes the URLs are even taken over by malicious or other websites, for example, for the TBDB entry with id 1356.

Similar database collections exist such as https://www.re3data.org, https://scicrunch.org, the Online Bioinformatics Resources Collection (https://www.hsls.pitt.edu/obrc/), or http://pathguide.org [8]. The Nucleic Acids Research (NAR) journal has gained popularity for its regular database issues publishing new or updated database resources in the life sciences [1]. A companion archive, the online molecular biology database collection, lists database summary records available at https://www.oxfordjournals.org/nar/database/a/. While this archive provides a good overview and the records appear to be updated yearly, several issues exist including but not limited to outdated or missing website links, no availability, and no revision information.

In this article, we describe a new web-based resource for the annotation of the NAR online molecular biology database collection. As the NAR collection provides only few information the primary goal was to enrich these entries, as well as to generate an up-to-date statistic of the availability and actuality of NAR-published databases. The relatively small size of the collection in comparison to Database Commons for example, makes it feasible to regularly update all entries and to provide automatic updates to the statistics.

## Materials and methods

Following the construction and contents of the website and database are described in detail.

### Base data collection

**Fig 1:**
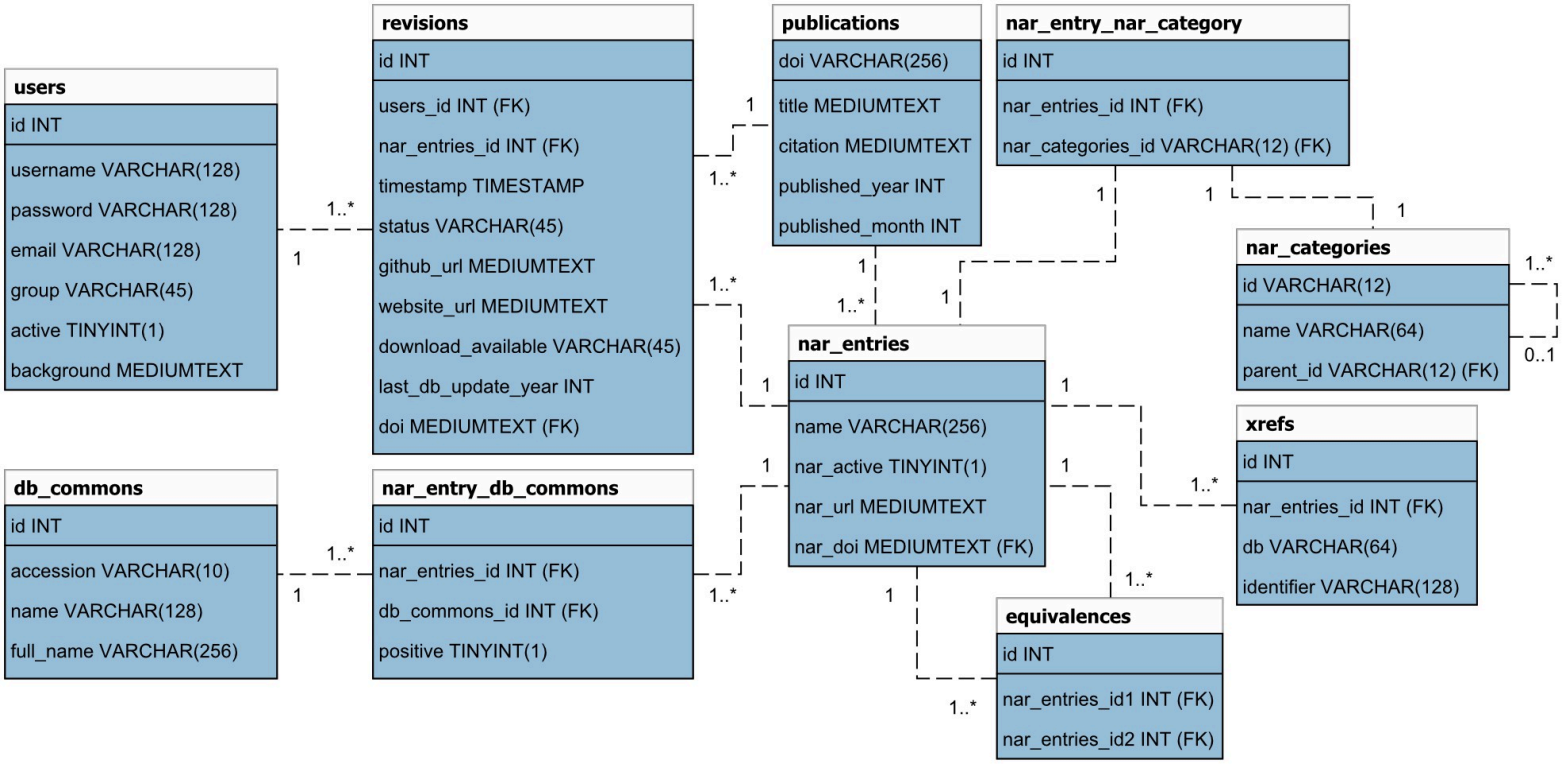
Database schema. Full relational database schema for NAR database entries, their annotations, and general website data.

The list of database entries and categories are extracted from the NAR database summary paper alphabetic list at https://www.oxfordjournals.org/nar/database/a/ and the category association list at https://www.oxfordjournals.org/nar/database/cap/. Each entry in this list contains a unique id, title, the authors, database URL, doi, and a short description. From these, the id, name, URL, and doi are extracted and stored as a table in a MySQL database. The categories, category-subcategory, and category-entry associations are stored in tables as well. The full schema for the website is visualized in Fig 1. Each publication doi in the database is enriched by retrieving the publication title, citation text, and publication year and month from the doi foundation API at http://dx.doi.org. As a basis for cross-references to Database Commons, the list of entry ids and names are retrieved from Database Commons and linked to the NAR database entries by simple name matching. All of these tasks are automated by PHP scripts enabling updates of the NAR information in the database with a single click in the admin backend. Whenever a new NAR database issue is published and their summary paper list is updated these scripts can be triggered. Database entries that are not present anymore in the summary list will have their “NAR active” flag set to false but remain available as a historic record.

### Website

For online availability as well as an annotation tool for the database entries a website is built using PHP, the Slim Framework version 4 (https://www.slimframework.com), and the Illuminate Database ORM library version 8 (https://github.com/illuminate/database). Bootstrap 5.3 (https://getbootstrap.com) is used for the general layout and Apache ECharts 5.4.2 [9] (https://echarts.apache.org) for statistical visualizations. Data storage and retrieval for the website use a standard MariaDB SQL database.

All database entries are accessible on the main page of the website. It is possible to search, sort, and filter the list of entries by several fields such as the name, status, last updated date, and more. Additionally, a category filter exists to search for database entries for specific topics. Each database entry has a details page with general information such as the publication’s doi, citation, website URLs, source code repository URLs, categories, as well as links to duplicate entries in the NAR database, external references, and the curation history. The curation history’s last entry always shows the latest information available in the NAR catalog. Above in descending order are the revisions saved by the manual curation process. Equivalent entries do not always share the same categories. Transitive categories from equivalent entries are listed as well with an asterisk and a small remark.

**Fig 2:**
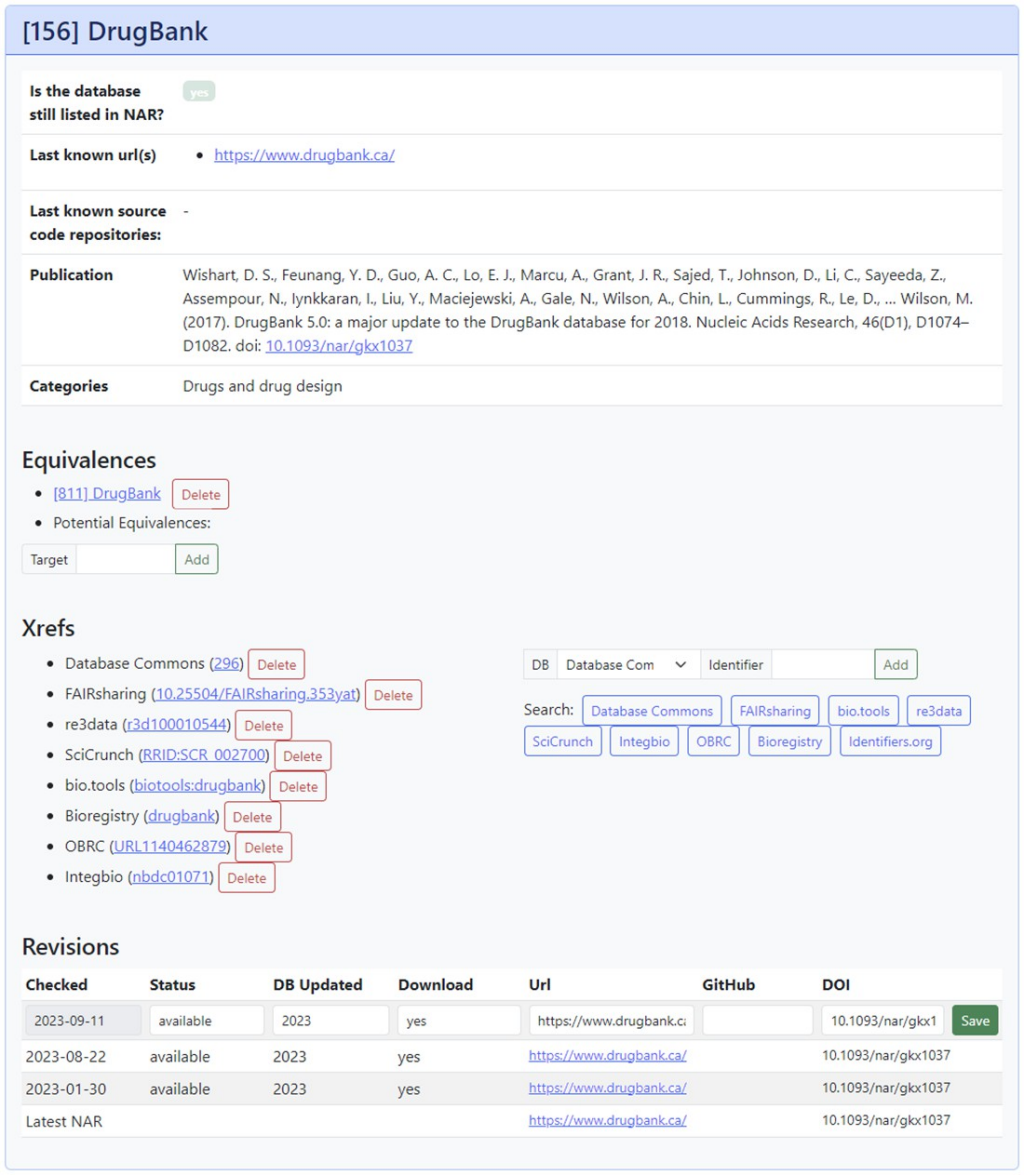
Entry editing mask screenshot. The user view of the database entry DrugBank with the existing information and the editing features available to logged-in users (modified to a light background for this publication).

Members of the annotation team are able to login and edit each entry on the respective details page such as updating the current status as well as adding or removing external references as shown in Fig 2. Direct user registration is not available at the moment to keep the annotation quality high and limit the risk of fraudulent behavior. However, interested users may request an account using the contact details on the website under “Feedback”. A statistics page is available with reports on different aspects of the database entries and the annotation process: The distribution of the database’s last updated year, download availability, status, curation staleness, and the entries count for each NAR category and subcategory. Visualizations for the statistics are implemented using the ECharts library. A tab-separated-values (TSV) file containing the latest annotations for all entries is available for download as well as a JSON endpoint for the timestamp of last modification.

### Data annotation

With the base NAR information in the database, the website is used to manually annotate each entry. The annotation mask is shown in Fig 2. First, the automatically generated potential equivalences based on entry names are checked for already annotated entries. Valid recommended equivalences or manually found ones that have no matching name can be added to the list. Second, the latest available database information is validated. This involves verifying that the website is still available, what is the newest version, and if the data can be downloaded. Additionally, the database website and publications are searched for links to source code repositories. All changed information is entered in the revision mask and saved. Third, cross-references to other database registries such as Database Commons are searched for and added using the xrefs mask. Helper buttons are available to quickly open the respective registry’s website search form with the current entries’ name as the search query. Whenever a new update is published to the NAR database collection, the annotation site administrator is able to automatically retrieve all changes.

## Results and discussion

The 2023 NAR database issue reports 1,764 databases in its database collection [1]. However, the base data acquisition retrieved 2,246 entries of which 2,025 are annotated to be unique. Duplicate entries in the NAR database collection exist because of multiple NAR publications about the same database or by pure duplication. An example of a pure duplication is the entries “IMG Genomes” (id 828) and “IMG/VR v3” (id 1945) both referencing the publication with doi “10.1093/nar/gkaa946”. While the database collection is already helpful, the provided information is limited. The presented web-based annotation platform allows the curation of these entries with minimal effort enriching the available information and gaining new insights into the database landscape. Additionally, the whole annotation history is transparent for each entry.

While limited to databases available in the NAR database collection only, the feasibility to maintain a relatively up-to-date curation state has been demonstrated with two active annotators at the time of writing. Other database registries either provide no last update date for their entries or the information has gone stale due to the entries not being updated regularly. Therefore, the provided recency of database annotations may better inform decision-making in database selection and provide more accurate statistics for a subset of the whole database landscape. The current number of 2,246 entries would require approximately six to be annotated every day for a full database update per year. With two annotators our current goal is set to annotate 20-40 entries per day actively annotating. This leaves room for days off, increases the update cycle for the whole database, as well as provides headroom for an increase in database entries over the years. In contrast to other database registries, we therefore guarantee our annotations to be continuously updated at least once a year.

**Fig 3:**
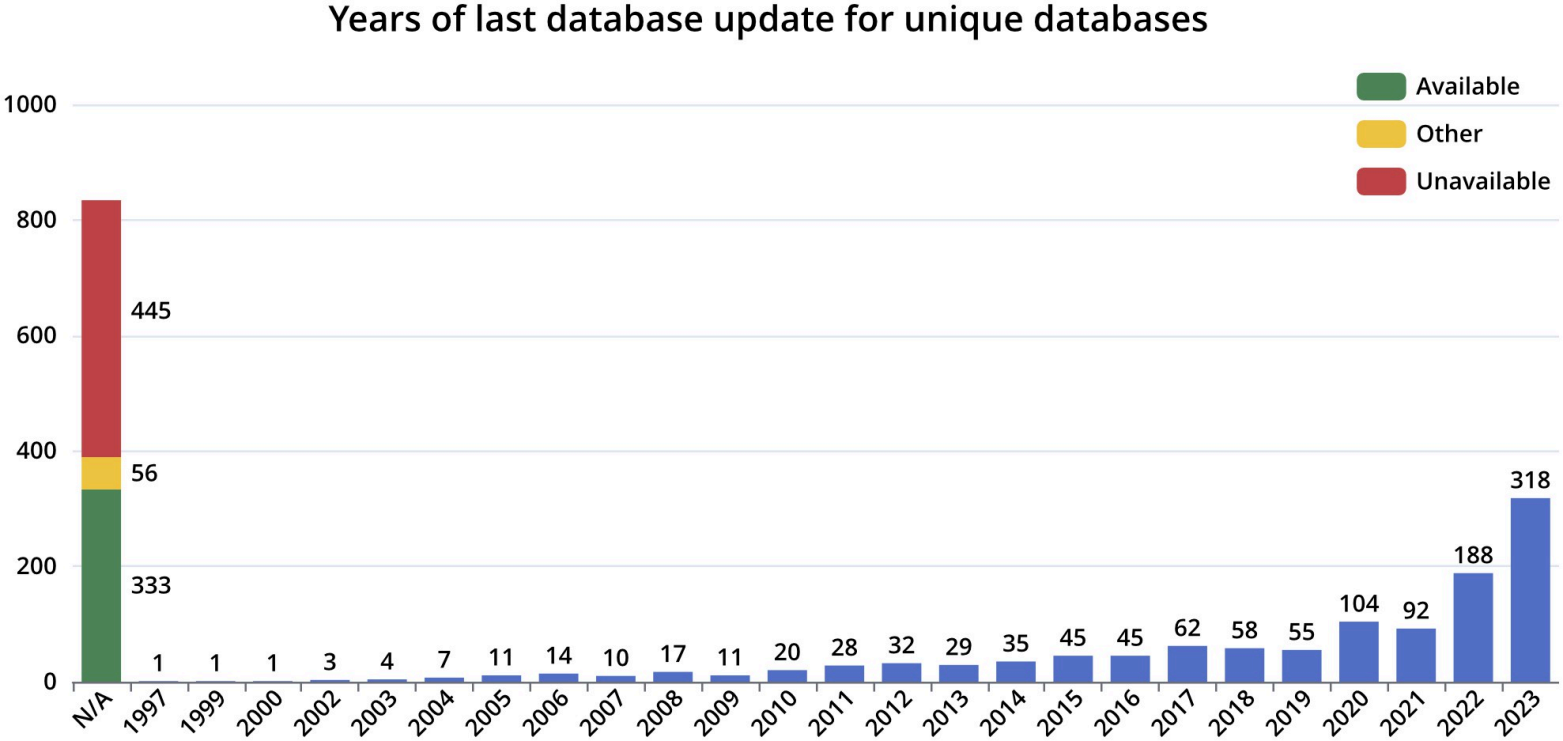
Database last update distribution chart. A bar plot visualizing the year of the last update for all database entries. If no information exists the number of available databases, not available, or have another status are visualized as the leftmost stacked bar in the plot.

The process of manual database curation has demonstrated several issues with many available databases. Most prominent is the lack of concise database revisions and update timestamps for many databases. While some clearly state a version number and release timestamp others only provide an update date in the text on some sub-page, in a downloaded files format, or may only be retrieved via FTP or ZIP file timestamps. Each of these workarounds introduces uncertainty about the validity of this information. A statistic for the years of the last update is visualized in Fig 3. This shows that many databases do not include this information at all or that the database is not available anymore. Most of the databases with known years are updated in the last four years.

**Fig 4:**
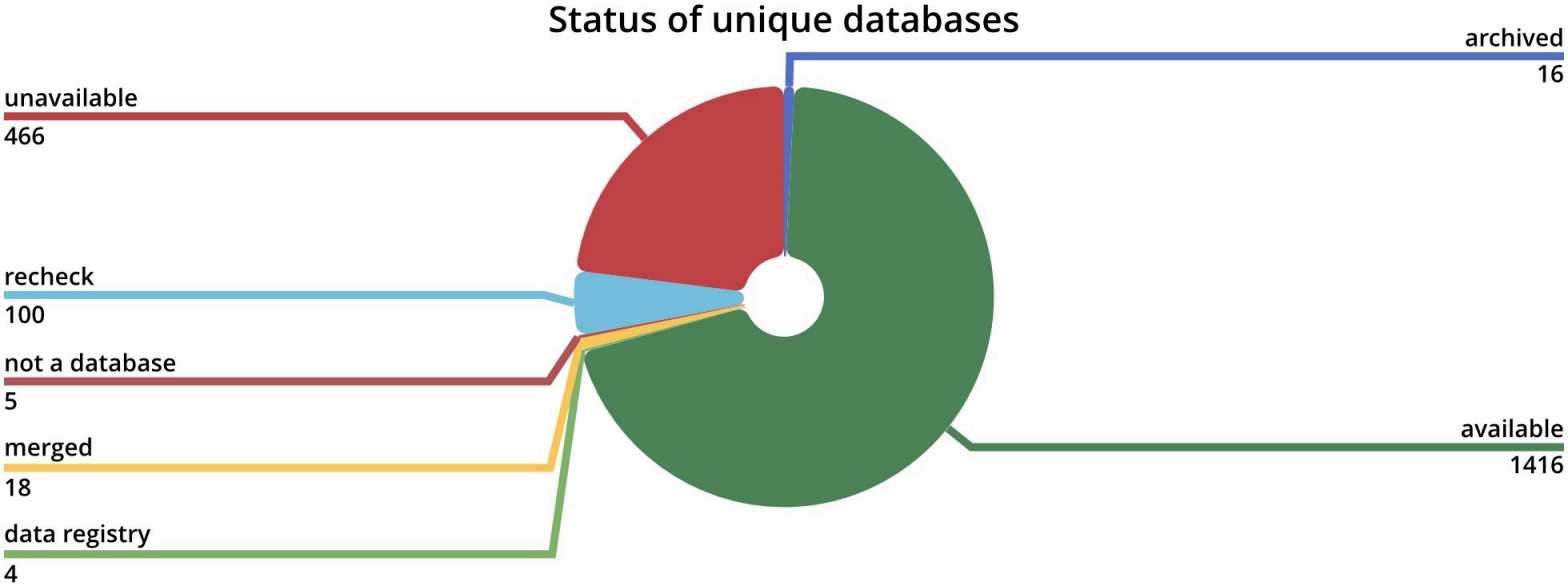
Database status distribution chart. A pie chart visualizing the status distribution of all unique databases.

Of all unique database entries, ∼75% are available online as visualized in Fig 4. According to Wren and Bateman in 2008 “approximately 20% of URLs published in MEDLINE abstracts are now inaccessible” [10] with is roughly in line with the results. Attwood *et al.* concluded in 2015 that “More than 60% of Web-based databases available in DBcat in 1997 have died” [11] differing significantly.

**Fig 5:**
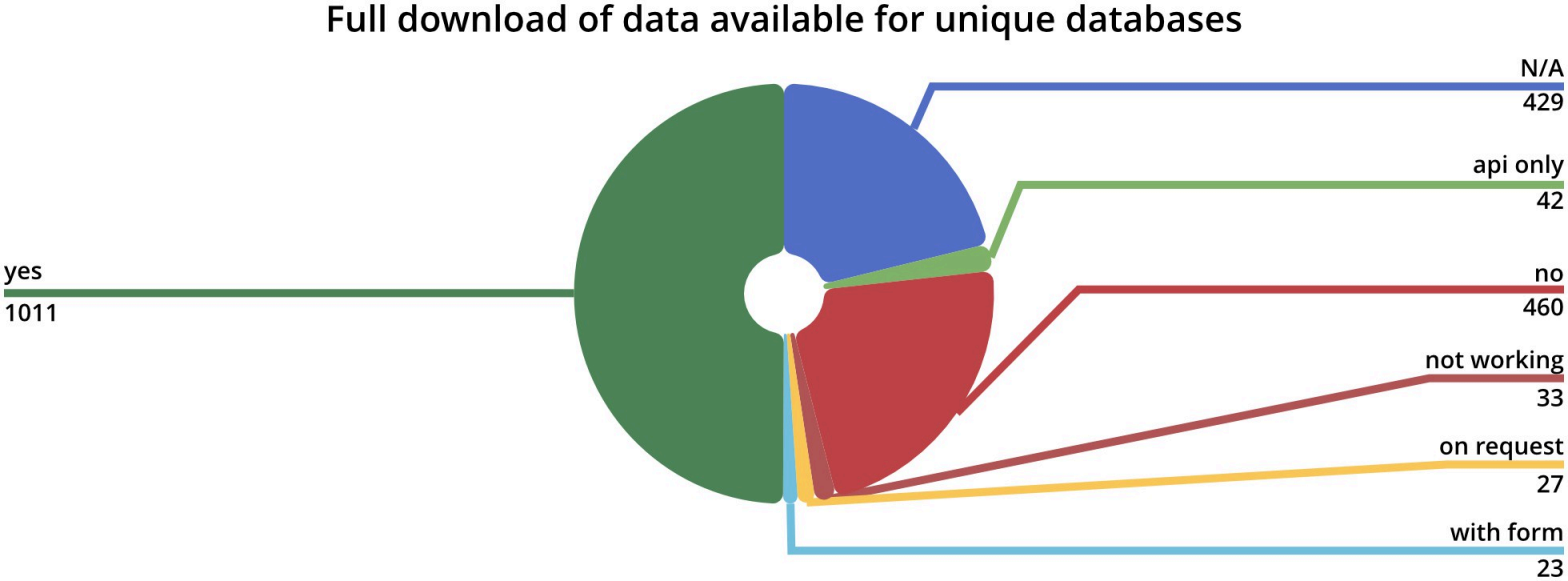
Database full download availability distribution chart. A pie chart visualizing the availability of full database downloads distribution of all unique databases.

Another statistic demonstrates, how many databases are downloadable as visualized in Fig 5. At least more than half is downloadable, however, for roughly 21% this information could not be determined. In combination the knowledge of which databases are up-to-date and can be downloaded greatly benefits database selection for research projects. To validate or audit database creation pipelines it has become more common under the FAIR principles to share source code repositories on platforms such as GitHub. Currently, 192 entries (159 unique databases) are annotated with links to source code repositories. The availability of this information may help databases gain even more trust in their quality.

Currently, 1,889 entries are cross-referenced to Database Commons either by automatic matching during base data creation or manual annotation. All automatically referenced entries were manually checked for false positives as some database names are abbreviations and not unique. This leaves 358 entries not cross-referenced as they are not included in Database Commons, for example, miRCarta [12] which is still available, was last updated in 2018, and the database can be downloaded. Even though Database Commons has many more database entries, the NAR database collection lists additional ones.

## Conclusion

The aim was to create a simple-to-use, web-based annotation platform for databases published in the NAR database collection and to provide up-to-date annotations for all entries. Currently, annotation information for the overall status, last date of a database update, categories, cross-references, links, and duplicates are available for each entry. In comparison to other databases, it provides additional information with links to source code repositories such as GitHub and whether a database is available for full download. Furthermore, the limited number of entries allows for the annotations to be of higher recency compared to registries such as Database Commons.

The latest annotations for all entries in the database are available for download in TSV format. Other database registries may use this data freely to update their existing entries or add entries not yet present in their database. A simple JSON endpoint can be used to retrieve the latest annotation timestamp to only update when new information has become available. This may further increase the overall quality of database metadata available online in general and potentially spark future collaborations.

The database is available online at https://nardbstatus.kalis-amts.de and has the potential to help in database selection and decision-making. It further provides a statistic on the current state of a subsection of all databases in the life sciences.
